# Loss-of-Function of *ATS1* Enhances Arabidopsis Salt Tolerance

**DOI:** 10.3390/plants12142646

**Published:** 2023-07-14

**Authors:** Yakun Liu, Guifen Wu, Xingxing Ke, Zhifu Zheng, Yueping Zheng

**Affiliations:** 1 The Key Laboratory for Quality Improvement of Agricultural Products of Zhejiang Province, College of Advanced Agricultural Sciences, Zhejiang A&F University, Hangzhou 311300, China; 15630712089@163.com (Y.L.); 13588701780@163.com (X.K.); zzheng@zafu.edu.cn (Z.Z.); 2 College of Forestry and Biotechnology, Zhejiang A&F University, Hangzhou 311300, China; wowcute@stu.zafu.edu.cn

**Keywords:** *Arabidopsis thaliana*, *ATS1*, salt tolerance

## Abstract

Despite the importance of lipid metabolism in various biological processes, little is known about the functionality of ATS1, a plastid glycerol-3-phosphate acyltransferase catalyzing the initial step of the prokaryotic glycerolipids biosynthetic pathway, in plant response to salt stress. In this study, both the loss-of-function mutants and the overexpression lines of *ATS1* were analyzed for salt tolerance properties. The results showed that *ATS1* overexpression lines had lower seed germination, shoot biomass, chlorophyll content, the proportion of relatively normal pod, and higher root/shoot ratio and anthocyanidin content compared with the wild type. Physiological and biochemical analysis revealed that *ats1* mutants had more unsaturated fatty acids to stabilize the plasma membrane under salt damage. Additionally, less induction of three main antioxidant enzymes activity and lower MDA content in *ats1* mutants indicated that mutation of the *ATS1* gene could reduce the damage extent. Furthermore, the *ats1* mutants maintained the K^+^/Na^+^ homeostasis by upregulating *HAK5* expression to increase K^+^ absorption and down-regulating *HKT1* expression to prevent Na^+^ uptake. This study suggested that the *ATS1* gene negatively affects salt resistance in Arabidopsis.

## 1. Introduction

Due to the increased anthropogenic activities and the changing natural environment, soil salinization caused by the accumulation of saline substances such as minerals and soluble salts in soil has become a serious problem [1]. According to statistics, 7.516 billion hectares of land are affected by salt globally in 831 nations, 75 of which have sizable tracts of saline soil [2]. As one of the most severe abiotic stresses, salt stress, causes almost 12 billion USD to be lost annually worldwide because of reduced agriculture production [3]. Identification and development of salt-tolerant germplasms or cultivars have become essential to prevent salinity damage to agricultural yield [4]. However, due to their complexity, many salt-tolerance mechanisms still remain to be comprehensively understood [5].

Plants have evolved numerous mechanisms to adapt to salinity, including ion homeostasis, osmolyte bio-synthesis, intracellular compartment of toxic ions, and the scavenging systems of reactive oxygen species (ROS) [6]. As a primary target of abiotic stresses, the plasma membrane serves as a biological barrier, separating the inside from the outside of the cell and forming specialized compartments to protect the contents of cells and organelles [7,8,9]. The fluidity and structure of the membrane, which are influenced by the degree of fatty acid desaturation, impact ATPase activity, bilayer permeability, and carrier-mediated transport [10]. Meanwhile, studies have shown that changes in the content of unsaturated fatty acids can affect plant tolerance to different environmental stresses such as cold, drought, heat, and salt by affecting the fluidity of the plasma membrane [11,12,13,14,15].

Glycerolipids are essential components of cell membranes and signaling molecules, participate in various physiological and biochemical processes, and play an essential role in plant growth and development [16]. Glycerol-3-Phosphate acyltransferase (GPAT) is a crucial enzyme in the glycerolipid synthetic pathway and is involved in different metabolic pathways and physiological functions [16]. It has been found that the *GPAT* genes are closely linked to fertility, stress tolerance, oil content of plants, and seed development [17]. Additionally, three different forms of GPAT, which are localized in the plastid, endoplasmic reticulum, and mitochondria, have been identified in plants [16]. There are ten known members of the Arabidopsis GPAT family, GPAT1–9, and soluble GPAT (ATS1). AtGPAT1/2/3 are found in the endoplasmic reticulum, AtGPAT4–9 are located in the mitochondria, and ATS1 is specifically located in the plastid.

ATS1 (Plastid glycerol-3-phosphate acyltransferase) is an essential enzyme that catalyzes the initial acylation of G3P (glycerol-3-phosphate) in the prokaryotic synthesis of glycerolipids [18]. ATS1 utilizes acyl-ACP as a substrate for acylation at the *sn*-1 position of G3P, generating LPA (lysophosphatidic acid). Subsequently, LPA is acylated by plastid lysophosphatidic acid acyltransferase (LPAAT1, also known as ATS2) to produce PA (phosphatidic acid) [18]. There have been many studies on the cloning and function of the *ATS1* gene in plastids. With the development of modern molecular biology, many genes homologous to the *ATS1* gene of *Arabidopsis thaliana* have been isolated and identified from plants such as *Pumpkin*, *Safflower*, *Cucumber*, *Sunflower*, and *Rape* [19,20,21]. The *ATS1* gene in these plants exhibits a variety of physiological functions and plays a vital role in plant growth, development, and stress resistance. When the *ATS1* of other species is heterologously overexpressed in Arabidopsis, the oil content in Arabidopsis seeds increases by 10–20% [22]. In contrast, inhibition of the activity of Arabidopsis *ATS1* resulted in a decrease in the oil content of Arabidopsis seeds by about 12% [18,22]. The *ats1* mutant significantly impacts the unsaturated fatty acids, which are essential constituents of cell membrane lipids, by reducing the content of C16:3 and increasing the content of C18:1, C18:2, and C18:3 in Arabidopsis leaves [23]. Spinach and Arabidopsis are more cold-resistant than squash due to their high unsaturated fatty acid content [24]. Furthermore, *BnATS1* promotes the accumulation of polyunsaturated fatty acids in cellular membranes, enhancing plant growth under low-temperature conditions in *Brassica napus* [18]. Overexpression of *LeATS1* can increase chilling tolerance in tomatoes by increasing the unsaturation of fatty acids of phosphatidylglycerol in the thylakoid membrane [25]. These researches suggest that the *ATS1* gene is involved in plant response to abiotic stress by modulating the unsaturation extent of the fatty acids.

At present, the researches on *ATS1* mainly focus on the effect on plants under chilling conditions. The role of the *ATS1* gene in the tolerance to salt stress requires further studies. Here, phenotypes of both loss-of-function mutants and overexpression Arabidopsis lines were investigated under normal and salt stress conditions. The results revealed that mutation of the *ATS1* gene improved salt tolerance by increasing the unsaturated fatty acid content, regulating the activity of antioxidant enzymes, and maintaining the K^+^/Na^+^ balance in Arabidopsis. Conversely, *ATS1* overexpression increased the sensitivity to salt stress. In conclusion, the *ATS1* gene played a negative role in response to salt stress. This work bridges a gap regarding the involvement of *ATS1* under salt stress and allows an in-depth analysis of the salt-induced regulation of *ATS1*.

## 2. Results

### 2.1. Overexpression and Mutation of ATS1 Produced No Visible Effect on Vegetative and Reproductive Growth of Arabidopsis thaliana under Normal Growth Conditions

**Figure 1:**
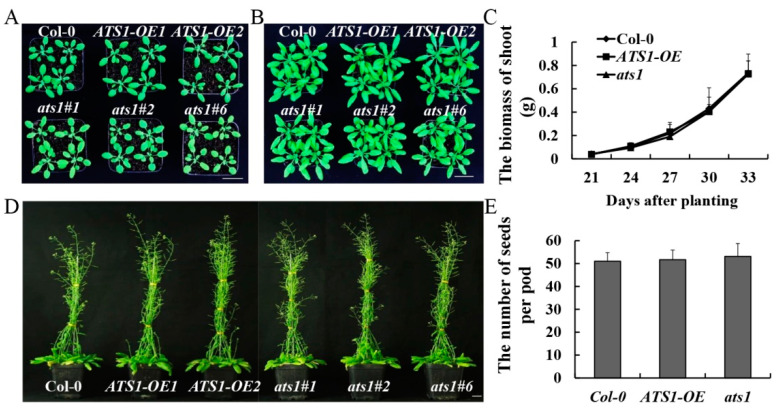
Phenotypic and physiological indexes analysis of Col-0, *ATS1-OE*, and *ats1* mutants under normal conditions. (**A**,**B**) Vegetative phenotypes were displayed at 24 days (**A**) and 30 days (**B**) after sowing in the soil. Bar = 2 cm. (**C**) Fresh shoot biomass was weighted from 21 to 33 days after sowing in soil. (**D**) Reproductive phenotypes were compared at 60 days after sowing in the soil. Bar = 2 cm. (**E**) Average number of seeds per pod statistics were counted.

Our laboratory has previously confirmed that *ATS1* gene is not necessary for plant growth and development under normal growth conditions [26]. However, it is not clear whether *ATS1* overexpression affects plant growth and development or not. Phenotypes were compared among *ATS1* overexpression (*ATS1-OE*), *ats1* mutant, and Col-0 (wild type) throughout the entire growth period to verify its effect. Under normal growth conditions, no noticeable differences in the aerial parts were observed among Col-0, mutants, and overexpressed lines during the vegetative stage (Figure 1A–C). Likewise, during the reproductive growth stage, the mutants and overexpressed lines showed similar bolting and number of pods with Col-0 (Figure 1D,E). The results indicated that *ATS1* was a nonessential gene in the normal growth and development of *Arabidopsis thaliana*.

### 2.2. Mutation of ATS1 Decreased Salt Sensitivity of Arabidopsis thaliana Seeds

**Figure 2:**
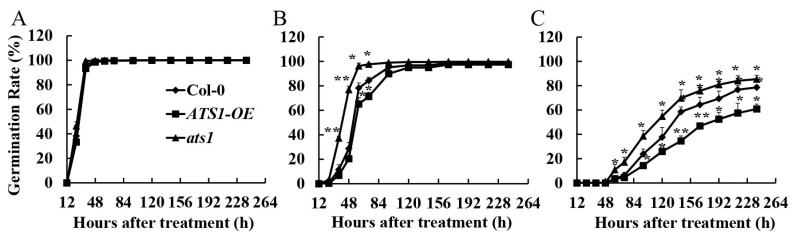
The germination rates of Col-0, *ATS1-OE*, and *ats1* mutants under 0 mM NaCl (**A**), 100 mM NaCl (**B**), and 200 mM NaCl (**C**) treatment in the 1/2 MS solid medium for 10 days. * and ** indicate the statistical significance compare with Col-0 at the levels of *p* ≤ 0.05 and *p* ≤ 0.01 (*n* = 3), respectively.

Seed germination, as a critical life stage for crops, is highly susceptible to the detrimental effects of salt stress [27]. High salinity levels have been shown to delay or even inhibit germination, leading to significant negative impacts on plant growth and crop production [28]. Seed germination rates of Col-0, *ats1* mutants, and overexpressed lines were determined under different treatments after 10 days. There was no significant difference in seed germination among the three genotypes under normal treatment (Figure 2A). However, after being exposed to 100 mM NaCl for 60 h, the germination rate of the *ats1* mutants reached nearly 100%, while that of Col-0 and overexpression lines were significantly lower. After 96 hours of treatment, the germination rate of the three genotypes almost reached 100% (Figure 2B). It suggested that the mutation of *ATS1* increased germination speed under the treatment of 100 mM NaCl. Compared with Col-0, the germination rate of the *ats1* mutants was significantly higher when the salt concentration reached 200 mM NaCl, while that of the *ATS1* overexpression lines was significantly lower. After being treated with 240 h, the germination rates of Col-0, *ATS1-OE*, and *ats1* mutant were 78.6%, 60.8%, and 85.39%, respectively (Figure 2C). These results indicated that the mutation of *ATS1* decreased the salt sensitivity of *Arabidopsis thaliana* seeds.

### 2.3. Overexpression of ATS1 Reduced the Salt Tolerance of Arabidopsis thaliana

**Figure 3:**
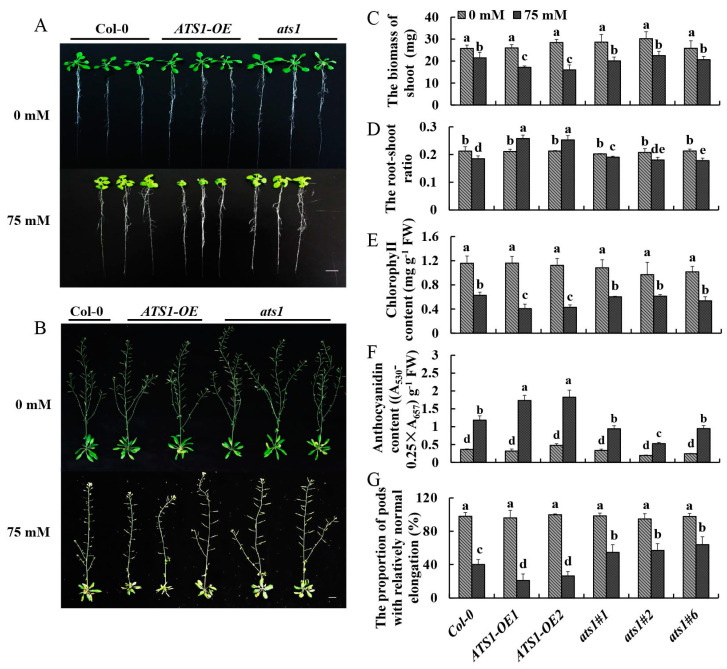
Phenotypic and physiological indexes of Col-0, *ATS1-OE,* and *ats1* mutants were compared under normal and salt stress conditions. (**A**) Phenotypes of seedlings were cultured in the 1/2 MS solid medium with or without 75 mM NaCl for 13 days. Bar = 2 cm. (**B**) Phenotypes of mature plants cultured in a hydroponic solution containing 0 or 75 mM NaCl for 7 weeks. Bar = 2 cm. (**C**–**F**) Determination of shoot biomass, root-to-shoot radio, chlorophyll content, and anthocyanin content in the 1/2 MS solid medium with or without 75 mM NaCl for 13 days. (**G**) Determination of the number of normal pods in a hydroponic solution containing 0 or 75 mM NaCl for 7 weeks. Data in (**C**–**G**) are shown as means ± SD (*n* = 3). For each column, different lowercase letters above the bars in (**C**–**G**) indicate a significant difference at *p* ≤ 0.05.

As one of the fundamental characteristics, the root–shoot ratio could be a parameter to access the overall physiological status of the analyzed genotypes [29]. And the chlorophyll content is directly related to the photosynthetic assimilation process in plants and serves as a physiological indicator to measure plant salt tolerance [30]. Additionally, anthocyanin is associated with the abiotic stress tolerance of plants, and its content increases significantly under various stress conditions [31]. For exploring the effects of the *ATS1* gene on vegetative and reproductive growth periods in plants under salt treatment, the above phenotypic indexes of Col-0, *ATS1-OE*, and *ats1* mutants were observed and determined under normal and salt treatments (Figure 3).

Phenotypes of these three genotypes without treatment showed no significantly different. Nevertheless, after being treated with 75 mM NaCl for 13 days in the 1/2 MS solid medium, the shoots’ growth was all inhibited, and the inhibition degree in overexpressed lines was the highest (Figure 3A). The aboveground biomasses of *ATS1-OE* were 20.1% and 25.5% smaller than that of the Col-0 under salt stress, respectively (Figure 3C). Moreover, *ATS1* overexpression increased the root-to-shoot ratio (Figure 3D). As the results showed, the chlorophyll contents of the leaves in *ATS1* overexpression lines decreased sharply under salt treatment and were 35.1% and 31.7% lower than that of Col-0 (Figure 3E). The anthocyanin contents of overexpressed lines were 46.4% and 54.3% higher than that of Col-0 with NaCl treatment (Figure 3F). However, the anthocyanin and chlorophyll concentrations of the leaves in *ats1* mutants showed no significantly different from those of Col-0. These findings suggested that overexpression of *ATS1* reduced the salt tolerance of Arabidopsis seedlings.

Similar to the period of vegetative growth, there was no discernible difference in pod elongation and seed setting among the three genotypes in the reproductive growth stage under normal treatment. As shown in Figure 3B, *ATS1* overexpression significantly hindered the growth of lateral branches and pods elongation under salt treatment. The number of normal pods in the *ATS1* overexpression lines was 47.5% and 33.7% lower than that of Col-0, while the value in the *ats1* mutants were 36.3%, 42.2%, and 59.4% greater (Figure 3G). In conclusion, the overexpression of *ATS1* reduced the salt tolerance of Arabidopsis in reproductive growth under salt stress.

### 2.4. ats1 Mutants Had Increased Unsaturated Fatty Acid Content under Salt Stress

Fatty acids are major components of membranes and contribute to the adaptation of cellular processes in response to environmental constraints [32]. Previous studies showed that the increase in unsaturated fatty acid content could enhance the resistance of plants to stress [33]. In our study, there was no significant difference in the total amounts of saturated and unsaturated fatty acids in leaves among three genotypes under normal treatment (Table 1). However, after salt treatment, the total unsaturated fatty acid content of the *ats1* mutants was higher than that of *ATS1-OE* and Col-0, while its total saturated fatty acid content was lower than that of both two (Table 2). These results suggested that the increased content of unsaturated fatty acid may play a vital role in enhancing salt tolerance of *ats1* mutants.

### 2.5. Overexpression Lines of ATS1 Were More Seriously Injured under Salt Stress

**Figure 4:**
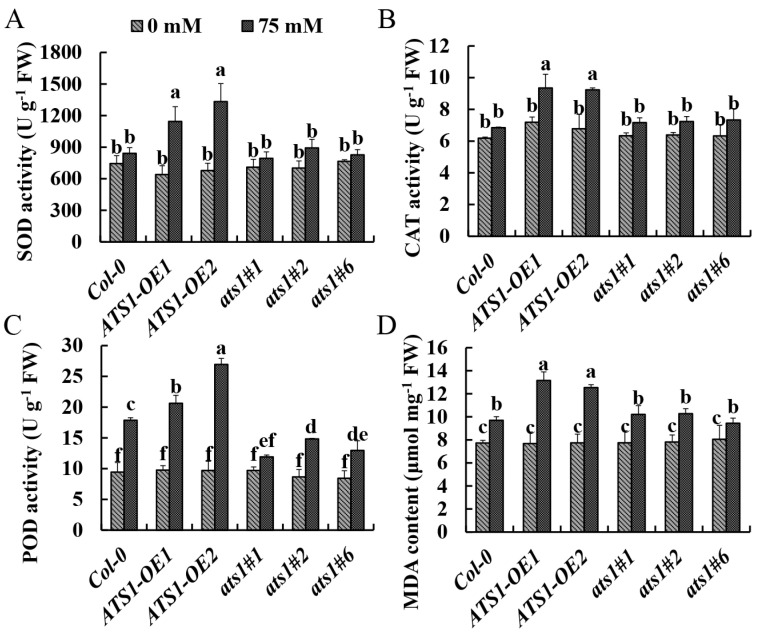
Effect of NaCl treatment on antioxidant enzyme activities and MDA content of Col-0, *ATS1-OE*, and *ats1* mutants. The activities of SOD (**A**), CAT (**B**), POD (**C**), and MDA content (**D**) in shoot were determined after being exposed to the 1/2 MS solid medium with or without 75 mM NaCl for 13 days. Data are shown as means ± SD (*n* = 3). Different lowercase letters above the bars indicate a significant difference at *p* ≤ 0.05 for each column.

Superoxide dismutase (SOD), peroxidase (POD), and catalase (CAT) are crucial enzymes involved in regulating reactive oxygen species (ROS) homeostasis [34,35]. They are vital regulators mediating signaling pathways involved in developmental processes and plant responses to environmental fluctuations [35]. Salt stress can induce the activity of these enzymes in plants, and the degree of induction increases with the severity of the stress [36]. MDA is an indicator of cell damage produced by lipid peroxidation, and the level of MDA reveals the sensitivity to salt stress and the extent of plant damage [36]. The present study showed that the activities of antioxidant enzymes and MDA content were similar in different genotypes under normal treatment. However, after 13 days of salt treatment, SOD and CAT activities of *ATS1* overexpressed plants increased significantly, while these values in Col-0 and *ats1* mutants showed no significant variations and were lower than those of *ATS1* overexpressed lines (Figure 4A,B). The POD activity of the three genotypes was all increased, but the increase in overexpressed lines was the highest (Figure 4C). The changes in the MDA content of three genotypes showed the same trends as those in POD activity (Figure 4D). Thus, the higher values of antioxidase activity and MDA content in *ATS1* overexpressed lines indicated that overexpression of *ATS1* could enhance the damage degree of plants under salt stress.

### 2.6. Mutation of ATS1 Maintained the Homeostasis of the K^+^/Na^+^ Ratio under Salt Stress

**Figure 5:**
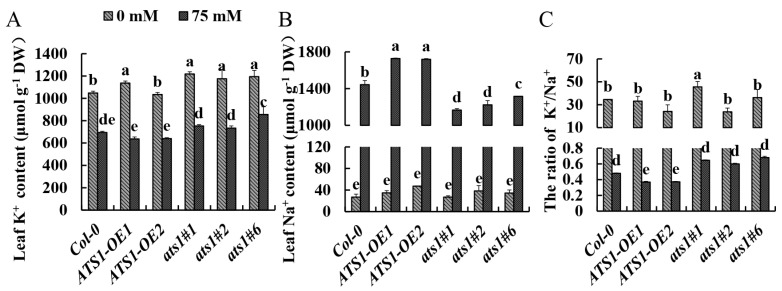
Accumulation of Na^+^ and K^+^ in the leaves of Col-0, *ATS1-OE,* and *ats1* mutants under salt stress. K^+^ content (**A**), Na^+^ content (**B**), and K^+^/Na^+^ ratio (**C**) were measured in the leaves of Col-0, *ATS1* overexpressed lines, and *ats1* mutants grown in hydroponic solution with or without 75 mM NaCl for 4 days. Data are shown as means ± SD (*n* = 3). Different lowercase letters above the bars indicate a significant difference at *p* ≤ 0.05 for each column.

K^+^ is essential for maintaining cell elasticity, membrane potential, membrane integrity and function, and the activity of many enzymes in different pathways. It is also a major cationic and essential cell nutrient [37]. Excessive Na^+^ in cells usually leads to K^+^ deficiency, and high concentration of Na^+^ eventually becomes toxic, disrupts ion homeostasis, and threats plant growth and development. It has been proposed that plant survival under salt stress requires a high cytosolic K^+^/Na^+^ ratio in the cytoplasm; maintaining a high K^+^/Na^+^ ratio under salt stress has become a crucial salt tolerance trait [38]. Under normal conditions, potassium and sodium content showed no significant difference among the three genotypes, and the K^+^/Na^+^ ratio in different genotypes remained consistent. Upon analysis of Na^+^ and K^+^ contents in the shoots, it was found that following 4-day salt stress, *ATS1-OE* plants had a lower K^+^ content compared to Col-0, while the average Na^+^ content was 19.66% higher than the Col-0 (Figure 5B). Consequently, the K^+^/Na^+^ ratio fell by 23.21% (Figure 5C). In contrast, the *ats1* mutants displayed a higher K^+^ content and K^+^/Na^+^ ratio (Figure 5A,C). This finding strengthened that the absence of *ATS1* may increase the salt tolerance by increasing the content of K^+^ to maintain the homeostasis of K^+^/Na^+^.

**Figure 6:**
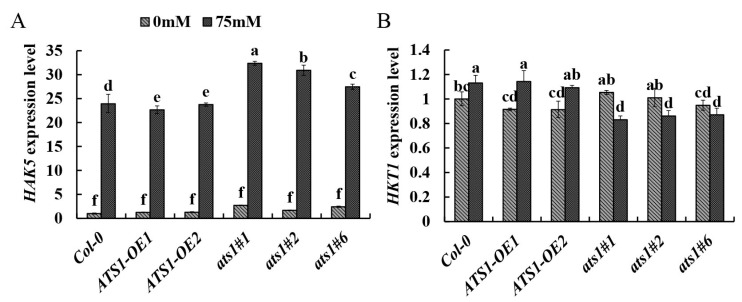
The relative expression of *HAK5* (**A**) and *HKT1* (**B**) gene in the root of Col-0, *ATS1* overexpressed lines and *ats1* mutants grown under control and 75 mM NaCl hydroponic solution for two days. Data are shown as means ± SD (*n* = 3). Different lowercase letters above the bars indicate a significant difference at *p* ≤ 0.05 for each column.

The high-affinity K^+^ transporter HAK5 is a main contributor to the uptake of root K^+^ from dilute solutions in K^+^-starved plants [39]. The Arabidopsis high-affinity K^+^ transporter 1 (HKT1) contributes to salinity tolerance by retrieving Na^+^ from the xylem stream to reduce its transport/accumulation to the shoots [40]. Therefore, we determined the expression level of *HAK5* and *HKT1* in three genotypes under control and salt treatments. The results showed that *HAK5* expressions were dramatically up-regulated in all genotypes under salt treatment. Particularly, *HAK5* expression in *ats1* mutants was significantly higher than that of the wild type, which improved K^+^ transportation and absorption to maintain the high K^+^ content in cells under salt treatment. Conversely, the expression level of *HAK5* in *ATS1* overexpression was lower than that of the wild type, which led to low content of K^+^ in the overexpression lines under salt treatment (Figure 6A). Under the control treatment, the *HKT1* expression showed no significant difference among the three genotypes. However, the *HKT1* expression level increased in *ATS1* overexpressed lines but decreased in *ats1* mutants after salt treatment, which resulted in increased Na^+^ accumulation in *ATS1* overexpressed lines and accelerated Na^+^ efflux in *ats1* mutants (Figure 6B). Therefore, these findings suggested that the *ats1* mutants improved plant salt tolerance by regulating cell ion content and maintaining ion homeostasis.

## 3. Discussion

Currently, there is still controversy about the role of the *ATS1* gene in plant growth and development. Kunst et al. found that seed growth and development of the *ats1* mutant were normal [41], while Xu et al. revealed that the reduction of *ATS1* RNA levels by RNAi led to decreased leaf size and inhibited seed set [42]. To further illuminate the function of the *ATS1* gene, we observed and compared the phenotypes of loss- and gain-of-function mutants. The results showed that both mutants’ vegetative and reproductive growth had no difference with Col-0 under normal conditions, suggesting that the *ATS1* is not necessary for plant growth and development (Figure 1). The emergence of opposite conclusions from previous studies might be due to the down-regulation of *ATS1* gene expression by RNAi interference technology, which may interfere with the function of other genes. However, the *ats1* mutants used in our study, obtained by CRISPR/Cas9 genome editing system, lost their function entirely, which suggested that CRISPR/Cas9 technology would help to understand gene function thoroughly. When treated with salt, *ats1* mutants showed more substantial resistance to stress, while overexpression lines had weaker stress resistance, indicating that the *ATS1* gene had a negative effect on plant salt tolerance (Figure 2 and Figure 3).

However, when treated with salt, *ats1* mutants showed more robust resistance to stress. At the same time, overexpression lines had weaker stress resistance, indicating that the *ATS1* gene had a negative effect on plant salt tolerance (Figure 2 and Figure 3). Plants have evolved several regulatory mechanisms, including physiological, biochemical, and morphological responses, to combat the harmful effects of salt stress [6]. Mutation of *ATS1* led to more unsaturated fatty acids in the plasma membrane, making the membrane less prone to phase transition at low temperatures and protecting the plant from cold stress [43]. Moreover, we observed an increased proportion of unsaturated fatty acid in *ats1* mutants and decreased proportion in overexpression lines, which might explain the differences in salt tolerance between these two genotypes (Table 2). The research on tomatoes strongly supported our conclusion, indicating that a high proportion of unsaturated fatty acids could improve salt tolerance [44]. Simultaneously, the higher induction of *HAK5* expression and suppression of *HKT1* expression was found in *ats1* mutants under the salt stress (Figure 6). High-affinity K^+^ transporter 5 (HAK5) belongs to the K^+^ transporter/high-affinity K^+^ transporter/K^+^ uptake protein (KUP/HAK/KT) family, one of the principal K^+^ acquisition systems in plants [45]. Furthermore, high-affinity potassium transporter 1 (HKT1), one of the Na^+^ transporters, has shown significant involvement in Na^+^ transport through its extrusion from circulation and recirculation [45]. Increasing K^+^ uptake and reducing Na^+^ uptake from high Na^+^ environments can improve plant salt tolerance by maintaining K^+^/Na^+^ homeostasis [46].

*ATS1* is involved in glycerolipid synthesis, with the prokaryotic pathway producing PA for synthesizing PG and galactolipids. In contrast, the eukaryotic pathway mainly produces PA for phospholipid synthesis and can be transported to the chloroplast for galactolipid synthesis [7]. These lipid molecules participate in and regulate various activities. For example, PA, as a second messenger, can rapidly accumulate under high salt, drought, and low-temperature stress to regulate stomatal aperture, cytoskeletal rearrangement, vesicle transport, and other cellular processes that regulate plant stress tolerance [47]. At the same time, PA promoted the synthesis of PG (phosphatidyl glycerol), DGDG (digalactosyl diglyceride), and MGDG (monogalactosyl diglyceride), which could improve plant salt tolerance [15]. However, the effect of *ATS1* on PA synthesis and the subsequent lipid metabolism under salt stress is not apparent. Therefore, further investigation is needed to understand how *ATS1* expression level affects salt stress in plants. 

## 4. Materials and Methods

### 4.1. Seed Sterilization

Arabidopsis Col-0, *ATS1* overexpression, and *ats1* mutant were used in the present study. The seeds were sterilized by being soaked in 75% (*v*/*v*) ethanol for 1 min [48], then in 35% (*v*/*v*) sodium hypochlorite and 0.05% Tween 20 for 5 min, followed by being washed three times with sterile distilled water. The seeds were kept dark and vernalized for 2–3 days.

### 4.2. Plasmid Construction and Plant Transformation

*ATS1* cDNA was obtained by RT-PCR of RNA isolated from Col-0 seedlings; the entire length of *ATS1* CDS was amplified by PCR using primers. Also, we obtained CAMV 35S promoter with *EcoR*I at the left end and NOS terminator with *Pst*I at the right end by PCR from pCAMBIA1305.1. We purified three PCR fragments and ligated them with the *EcoR*I/*Pst*I-linearized binary pCAMBIA1300 using seamless cloning and assembly kit, resulting in the P35S:*ATS1* recombinant plasmid. The constructed plasmids were introduced into *Agrobacterium tumefaciens* strain GV3101 and transformed into plants using the floral dip method. Transgenic plants were selected by hygromycin and timentin resistance, and T_3_ homozygotes were used for all analyses. The primers used during plasmid construction are listed in Table A1 in Appendix A. Meanwhile, the CRISPR/Cas9 system was used to generate *ats1* mutants [49].

### 4.3. Germination of Seeds and Growth Conditions

The sterilized and vernalized seeds were uniformly scattered on 1/2 Murashige and Skoog Basal Medium (MS) round plant medium with different concentrations of NaCl (0, 100, and 200 mM) and cultured at 22 °C with 16 h light/8 h darkness cycle, 40% humidity. The germination rate was calculated every 12 h for ten days, with germination being determined when the Arabidopsis radicle penetrated the seed coat.

Germination rate (%) = Total number of germinated seeds in test time (day)/number of seeds tested × 100.

### 4.4. Determination of Chlorophyll and Anthocyanin Content

(1) Chlorophyll content: Thirty mg of leaves from different genotypes were weighed and placed into separate glass centrifuge tubes. Then, 4 mL of 80% (*v*/*v*) acetone was added to each tube. The tubes were shaken vigorously to ensure the leaves were thoroughly soaked in the solution. The tubes were placed at 4 °C and in darkness overnight. After 24 h of treatment, the absorbance at 645 nm and 663 nm were measured using a spectrophotometer, and the chlorophyll content was calculated using the following formula:

Chl a (mg/L) = 12.7A_663_ − 2.69A_645_ (1)

Chl b (mg/L) = 22.9A_645_ − 4.68A_663_ (2)

Ct (total chlorophyll content) = Chl a + Chl b (mg/L) = 8.02A_663_ + 20.21A_645_ (3)

(2) Anthocyanin content: Thirty mg of leaves from different genotypes were weighed and placed into separate glass centrifuge tubes. Then, 4 mL of 1% (*v*/*v*) hydrochloric acid/methanol solution was added to each tube. The tubes were shaken vigorously to ensure the leaves were thoroughly soaked in the solution. These tubes were placed at 4 °C and in darkness overnight. After 24 h of treatment, the absorbance at 530 nm and 657 nm was measured using a spectrophotometer, (A_530_ − 0.25 × A_657_) per gram fresh weight was used to quantify the relative amount of anthocyanins [50].

### 4.5. Determination of Fatty Acid Composition

The fatty acid components of Arabidopsis leaves were determined by the method described in the literature by Cahoon et al. [51]. Five biological replicates were set for each line. Agilent 7890B gas chromatograph was used in this experiment. The content of fatty acid components was calculated by peak area integral method.

### 4.6. Determination of Antioxidant Enzymes Activities and MDA Content

The plants were grown on 1/2 MS medium for 4 days before being transferred to 75 mM NaCl medium for 13 days; the shoots were frozen at −80 °C for subsequent analysis. SOD activity was determined by the nitrogen blue tetrazole method [52], CAT activity by the visible light method, and POD activity by the guaiacol method [53]. The amount of MDA was determined by colorimetric method based on TBA [54]. Repeat each treatment at least 3 times (the experiments were conducted on ice for one day to avoid environmental effects on enzyme activity). Three biological replicates were set for each strain.

### 4.7. Determination of K^+^ and Na^+^ Content

The plants were cultivated in 1/5 Hoagland Solution for 3 weeks, treated with 75 mM NaCl hydroponic solution for 4 days, and the leaves were rinsed with ionized water and dried at 70 °C for over 6 h. Fifty mg samples were taken and ground, then 7 mL concentrated nitric acid and 1 mL 30% hydrogen peroxide aqueous solution were added [55]. The mix was placed at room temperature for 2 h before being digested by microwave digestion instrument. The contents of K^+^ and Na^+^ were determined by atomic absorption spectrometry. Three biological replicates were set for each line. The calculation formula of element contents is as follows:

C = [(Cn – C’) × 0.015/(M × 10^−3^)]/m (4)

Note: C: The concentration of the ion per g dry weight of plant samples, unit: μmol g^−1^ DW; Cn: The concentration of the ion in each sample digestion solution, unit: mg L^−1^; C’: The concentration of the ion in blank control digestion solution, unit: mg L^−1^; M: The relative molecular weight of the element to be measured, unit: g mol^−1^; m: Dry weight of the sample, units: g.

### 4.8. Expression Analysis of Related Genes

The plants were cultivated in 1/5 Hoagland Solution for 3 weeks. After being treated with salt stress for 2 days, the roots of three genotypes from control and salt treatments were obtained. To analyze the expression level of *HAK5* and *HKT1*, total RNAs of the root material were extracted using Trizol reagent (TransGen Biotech, Bejing, China) according to the manufacturer’s protocols. Total RNAs were converted into cDNAs using Hifair Ⅱ 1st Strand cDNA Synthesis SuperMix (Yeasen, Shanghai, China). Quantitative real-time PCR (qRT-PCR) analysis used the Hieff qPCR SYBR Green Master Mix (Yeasen, Shanghai, China). The 2^−ΔΔCt^ method was used to analyze the experimental data. The primers are listed in Table A1.

### 4.9. Data Processing and Analysis

Excel 2016 software was used for data statistics and plotting. Analyses of statistics analysis of variance (ANOVA) was used to examine statistical differences between measures taken on several days and during various treatments. When differences reached a probability level of *p* ≤ 0.05, they were deemed significant. These analyses were conducted using the SPSS software (Chicago, IL, USA).

## 5. Conclusions

This study demonstrated that *ATS1* is not necessary for the normal growth and development of plants. It also revealed that the mutation of *ATS1* enhanced salt tolerance by improving the unsaturated fatty acid content and maintaining the K^+^/Na^+^ homeostasis. This study focuses on the expression of *ATS1* in the degree of membrane lipid unsaturation at different salinities. It is expected to provide a new idea for in-depth exploration of the further mechanism in plant salt stress resistance and the relationship between lipid function. However, lipid signaling during this period is not mentioned. Moreover, the molecular mechanism of the *ATS1* gene regulating plant salt stress response remains to be further studied.

## Figures and Tables

**Table 1:** Determination of the fatty acid composition (%) of membrane lipids under normal treatment.

Fatty Acid Composition	Col-0	*ATS1-OE1*	*ATS1-OE2*	*ats1#1*	*ats1#2*	*ats1#6*
C14:0	0.50 ± 0.03	0.52 ± 0.05	0.56 ± 0.07	0.49 ± 0.05	0.48 ± 0.03	0.48 ± 0.08
C14:1	1.27 ± 0.05	1.22 ± 0.11	1.43 ± 0.13	1.23 ± 0.21	1.26 ± 0.21	1.32 ± 0.16
C16:0	18.37 ± 0.2	21.38 ± 2.42	21.20 ± 0.56	17.91 ± 1.24	16.78 ± 1.24	16.21 ± 0.99
C16:1	3.28 ± 0.1	3.74 ± 0.2	3.66 ± 0.1	2.37 ± 0.12	2.49 ± 0.12	2.49 ± 0.08
C16:3	10.85 ± 0.2	12.91 ± 027	12.02 ± 0.18	0.86 ± 0.03	0.63 ± 0.03	0.67 ± 0.12
C18:0	1.97 ± 0.11	3.42 ± 0.3	2.94 ± 0.53	2.51 ± 0.43	2.84 ± 0.43	2.56 ± 0.53
C18:1cis	3.63 ± 0.2	3.30 ± 0.15	3.33 ± 0.2	8.29 ± 0.15	8.06 ± 0.15	8.09 ± 0.14
C18:2cis	16.19 ± 0.23	13.89 ± 0.8	15.24 ± 0.36	18.90 ± 0.73	18.73 ± 0.73	18.92 ± 0.67
C18:3n3	43.50 ± 0.13	39.75 ± 2.3	39.83 ± 0.82	47.81 ± 1.6	48.41 ± 1.6	48.79 ± 1.86
Saturated fatty acid	20.82 ± 0.26	21.34 ± 0.75	21.51 ± 0.9	21.18 ± 0.68	21.34 ± 0.42	20.34 ± 0.92
Unsaturated fatty acid	79.18 ± 0.26	78.66 ± 0.75	78.49 ± 0.9	78.82 ± 0.68	78.66 ± 0.42	79.66 ± 0.92

Note: Data in Table 1 are shown as means ± SD (*n* = 3).

**Table 2:** Determination of the fatty acid composition (%) of membrane lipids under salt treatment.

Fatty Acid Composition	Col-0	*ATS1-OE1*	*ATS1-OE2*	*ats1#1*	*ats1#2*	*ats1#6*
C14:0	0.57 ± 0.05	0.50 ± 0.03	0.51 ± 0.02	0.62 ± 0.11	0.74 ± 0.1	0.68 ± 0.05
C14:1	1.52 ± 0.19	1.40 ± 0.09	1.44 ± 0.12	1.36 ± 0.19	1.66 ± 0.14	1.47 ± 0.09
C16:0	21.54 ± 1.25	20.81 ± 0.24	21.41 ± 0.83	17.43 ± 1.37	15.30 ± 1.07	14.13 ± 0.57
C16:1	3.19 ± 0.1	3.52 ± 0.04	3.57 ± 0.11	2.73 ± 0.26	2.40 ± 0.11	2.41 ± 0.1
C16:3	10.19 ± 0.55	12.42 ± 0.16	11.72 ± 0.28	0.86 ± 0.07	0.55 ± 0.04	0.52 ± 0.02
C18:0	3.67 ± 1.16	2.07 ± 0.1	2.95 ± 0.33	3.96 ± 0.91	4.01 ± 1.07	2.79 ± 0.14
C18:1cis	2.84 ± 0.08	2.81 ± 0.08	2.92 ± 0.27	5.58 ± 0.17	6.74 ± 0.16	7.02 ± 0.14
C18:2cis	15.46 ± 0.77	13.56 ± 0.13	14.48 ± 0.68	20.85 ± 0.8	20.56 ± 0.61	20.50 ± 0.33
C18:3n3	43.09 ± 2.61	42.91 ± 0.18	41.50 ± 0.71	46.42 ± 0.31	48.86 ± 0.53	50.58 ± 0.74
Saturated fatty acid	25.95 ± 1.15 **a**	23.38 ± 0.2 **b**	25.06 ± 1.27 **a**	17.72 ± 0.93 **c**	18.85 ± 0.63 **c**	17.49 ± 0.63 **c**
Unsaturated fatty acid	74.05 ± 1.15 **c**	76.62 ± 0.2 **b**	74.94 ± 1.27 **c**	82.28 ± 0.93 **a**	81.15 ± 0.63 **a**	82.51 ± 0.63 **a**

Note: Data in Table 2 are shown as means ± SD (*n* = 3). Different lowercase letters indicate a significant difference at *p* ≤ 0.05 for each row.

## Data Availability

All data generated or analyzed during this study are included in this published article.

## Appendix A

**Table A1:** Sequences of primers used in this study.

Primer	Sequence (5′-3′)
AtHAK5-FP	TCTGCATCACTGGGACGGAG
AtHAK5-RP	CAGTATAACGGATCAGGGATTGA
AtHKT1-FP	CAAGTGGTGAATTCGCGACA
AtHKT1-RP	CATCATCTCCTCCTTCTTTCTCT
ATS1 PCR-FP	ATGACTCTCACGTTTTCCTC
ATS1 PCR-RP	CTAATTCCAAGGTTGTGACA
CAMV 35S promotor FP	TATGACCATGATTACGAATTCCATGGAGTCAAAGATTCAAA
CAMV 35S promotor RP	AAACGTGAGAGTCATAGTCCCCCGTGTTCTCTCCA
NOS terminator FP	CAACCTTGGAATTAGCGTTCAAACATTTGGCAATA
NOS terminator RP	GCCAAGCTTGCATGCCTGCAGCCCGATCTAGTAACATAGAT

## References

[1] Lei G., Zeng W., Jiang Y., Ao C., Wu J., Huang J. (2021). Sensitivity analysis of the SWAP (Soil-Water-Atmosphere-Plant) model under different nitrogen applications and root distributions in saline soils. Pedosphere.

[2] Amini S., Ghadiri H., Chen C., Marschner P. (2016). Salt-affected soils, reclamation, carbon dynamics, and biochar: A review. J. Soils Sediments.

[3] Behera T.K., Krishna R., Ansari W.A., Aamir M., Kumar P., Kashyap S.P., Pandey S., Kole C. (2021). Approaches involved in the vegetable crops salt stress tolerance improvement: Present status and way ahead. Front. Plant Sci..

[4] Munns R. (2005). Genes and salt tolerance: Bringing them together. New Phytol..

[5] Wani S.H., Kumar V., Khare T., Guddimalli R., Parveda M., Solymosi K., Suprasanna P., Kavi Kishor P.B. (2020). Engineering salinity tolerance in plants: Progress and prospects. Planta.

[6] Zhao S., Zhang Q., Liu M., Zhou H., Ma C., Wang P. (2021). Regulation of plant responses to salt stress. Int. J. Mol. Sci..

[7] Rawat N., Singla-Pareek S.L., Pareek A. (2021). Membrane dynamics during individual and combined abiotic stresses in plants and tools to study the same. Physiol. Plant..

[8] Guo Q., Liu L., Barkla B.J. (2019). Membrane lipid remodeling in response to salinity. Int. J. Mol. Sci..

[9] Watson H. (2015). Biological membranes. Essays Biochem..

[10] Sui N., Tian S.S., Wang W.Q., Wang M.J., Fan H. (2017). Overexpression of glycerol-3-phosphate acyltransferase from *Suaeda salsa* improves salt tolerance in Arabidopsis. Front. Plant Sci..

[11] Dakhma W.S., Zarrouk M., Cherif A. (1995). Effects of drought-stress on lipids in rape leaves. Phytochemistry.

[12] Olsson M. (1995). Alterations in lipid composition, lipid peroxidation and anti-oxidative protection during senescence in drought stressed plants and non-drought stressed plants of pisum sativum. Plant Physiol. Biochem..

[13] Matos M.C., Campos P.S., Ramalho J.C., Marques N.M., Matos A. (2002). Photosynthetic activity and cellular integrity of the andean legume *Pachyrhizus ahipa* (Wedd.) parodi under heat and water stress. Photosynthetica.

[14] Liu X.Y., Li B., Yang J.H., Sui N., Yang X.M., Meng Q.W. (2008). Overexpression of tomato chloroplast omega-3 fatty acid desaturase gene alleviates the photoinhibition of photosystems 2 and 1 under chilling stress. Photosynthetica.

[15] Sui N., Han G. (2014). Salt-induced photoinhibition of PSII is alleviated in halophyte *Thellungiella halophila* by increases of unsaturated fatty acids in membrane lipids. Acta Physiol. Plant..

[16] Chen X., Snyder C.L., Truksa M., Shah S., Weselake R.J. (2011). sn-Glycerol-3-phosphate acyltransferases in plants. Plant Signal. Behav..

[17] Payá-Milans M., Aznar-Moreno J.A., Balbuena T.S., Haslam R.P., Gidda S.K., Pérez-Hormaeche J., Mullen R.T., Thelen J.J., Napier J.A., Salas J.J. (2016). Sunflower *Ha*GPAT9-1 is the predominant GPAT during seed development. Plant Sci..

[18] Kang H., Jia C., Liu N., Aboagla A.A.A., Chen W., Gong W., Tang S., Hong Y. (2020). Plastid glycerol-3-phosphate acyltransferase enhanced plant growth and prokaryotic glycerolipid synthesis in *Brassica napus*. Int. J. Mol. Sci..

[19] Nishida I., Sugiura M., Enju A., Nakamura M. (2000). A second gene for acyl-(acyl-carrier-protein): Glycerol-3-phosphate acyltransferase in squash, Cucurbita moschata cv. Shirogikuza(*), codes for an oleate-selective isozyme: Molecular cloning and protein purification studies. Plant Cell Physiol..

[20] Bhella R.S., MacKenzie S.L. (1994). Nucleotide sequence of a cDNA from Carthamus tinctorius encoding a glycerol-3-phosphate acyl transferase. Plant Physiol..

[21] Payá-Milans M., Venegas-Calerón M., Salas J.J., Garcés R., Martínez-Force E. (2015). Cloning, heterologous expression and biochemical characterization of plastidial sn-glycerol-3-phosphate acyltransferase from Helianthus annuus. Phytochemistry.

[22] Misra A., Khan K., Niranjan A., Kumar V., Sane V.A. (2017). Heterologous expression of two GPATs from *Jatropha curcas* alters seed oil levels in transgenic *Arabidopsis thaliana*. Plant Sci..

[23] Kunst L., Browse J., Somerville C. (1988). Altered regulation of lipid biosynthesis in a mutant of Arabidopsis deficient in chloroplast glycerol-3-phosphate acyltransferase activity. Proc. Natl. Acad. Sci. USA.

[24] Murata N., Ishizaki-Nishizawa O., Higashi S., Hayashi H., Tasaka Y., Nishida I. (1992). Genetically engineered alteration in the chilling sensitivity of plants. Nature.

[25] Sun Y.L., Li F., Su N., Sun X.L., Zhao S.J., Meng Q.W. (2010). The increase in unsaturation of fatty acids of phosphatidylglycerol in thylakoid membrane enhanced salt tolerance in tomato. Photosynthetica.

[26] Ke X.X., Liu Y.K., Xu X.Z., Lin Y.X., Zheng Z.F., Zheng Y.P. (2023). Loss-of-function mutations in *ATS1* reveal its dispensable role in normal seed development of Arabidopsis. J. Zhejiang A&F Univ..

[27] Hajihashemi S., Skalicky M., Brestic M., Pavla V. (2020). Cross-talk between nitric oxide, hydrogen peroxide and calcium in salt-stressed Chenopodium quinoa Willd. At seed germination stage. Plant Physiol. Biochem..

[28] Ibrahim E.A. (2016). Seed priming to alleviate salinity stress in germinating seeds. J. Plant Physiol..

[29] Bláha L. (2019). Importance of root—Shoot ratio for crops roduction. J. Agron. Agric. Sci..

[30] Fleischer W.E. (1935). The relation between chlorophyll content and rate of photosynthesis. J. Gen. Physiol..

[31] Huot B., Yao J., Montgomery B.L., He S.Y. (2014). Growth–defense tradeoffs in plants: A balancing act to optimize fitness. Mol. Plant.

[32] Huang J., Xue C., Wang H., Wang L., Schmidt W., Shen R., Lan P. (2017). Genes of ACYL CARRIER PROTEIN family show different expression profiles and overexpression of ACYL CARRIER PROTEIN 5 modulates fatty acid composition and enhances salt stress tolerance in *Arabidopsis*. Front. Plant Sci..

[33] Mikami K., Murata N. (2003). Membrane fluidity and the perception of environmental signals in cyanobacteria and plants. Prog. Lipid Res..

[34] Wang J., Chen G., Zhang C. (2002). The effects of water stress on soluble protein content, the activity of SOD, POD and CAT of two ecotypes of reeds (Phragmites communis). Acta Bot. Bras..

[35] Wang C.T., Ru J.N., Liu Y.W., Li M., Zhao D., Yang J.F., Fu J.D., Xu Z.S. (2018). Maize WRKY transcription factor *Zm*WRKY106 confers drought and heat tolerance in transgenic plants. Int. J. Mol. Sci..

[36] Baxter A., Mittler R., Suzuki N. (2014). ROS as key players in plant stress signalling. J. Exp. Bot..

[37] Ashley M.K., Grant M., Grabov A. (2006). Plant responses to potassium deficiencies: A role for potassium transport proteins. J. Exp. Bot..

[38] Shabala S., Pottosin I. (2014). Regulation of potassium transport in plants under hostile conditions: Implications for abiotic and biotic stress tolerance. Physiol. Plant..

[39] Lara A., Ródenas R., Andrés Z., Martínez V., Quintero F.J., Nieves-Cordones M., Botella M.A., Rubio F. (2020). Arabidopsis K^+^ transporter HAK5-mediated high-affinity root K^+^ uptake is regulated by protein kinases CIPK1 and CIPK9. J. Exp. Bot..

[40] Ali A., Maggio A., Bressan R.A., Yun D.J. (2019). Role and functional differences of HKT1-type transporters in plants under salt stress. Int. J. Mol. Sci..

[41] Kunst L., Browse J., Somerville C. (1989). Altered chloroplast structure and function in a mutant of Arabidopsis deficient in plastid glycerol-3-phosphate acyltransferase activity. Plant Physiol..

[42] Xu C., Yu B., Cornish A.J., Froehlich J.E., Benning C. (2006). Phosphatidylglycerol biosynthesis in chloroplasts of Arabidopsis mutants deficient in acyl-ACP glycerol-3- phosphate acyltransferase. Plant J..

[43] Xue M., Guo T., Ren M., Wang Z., Tang K., Zhang W., Wang M. (2019). Constitutive expression of chloroplast glycerol 3-phosphate acyltransferase from *Ammopiptanthus mongolicus* enhances unsaturation of chloroplast lipids and tolerance to chilling, freezing and oxidative stress in transgenic Arabidopsis. Plant Physiol. Biochem..

[44] Sui N., Li M., Zhao S.J., Li F., Liang H., Meng Q.W. (2007). Overexpression of glycerol-3-phosphate acyltransferase gene improves chilling tolerance in tomato. Planta.

[45] Assaha D.V.M., Ueda A., Saneoka H., Al-Yahyai R., Yaish M.W. (2017). The role of Na^+^ and K^+^ transporters in salt stress adaptation in glycophytes. Front. Physiol..

[46] Shi H., Lee B.H., Wu S.J., Zhu J.K. (2003). Overexpression of a plasma membrane Na^+^/H^+^ antiporter gene improves salt tolerance in *Arabidopsis thaliana*. Nat. Biotechnol..

[47] Zhang Q., Xiao S.Y. (2015). Lipids in salicylic acid-mediated defense in plants: Focusing on the roles of phosphatidic acid and phosphatidylinositol 4-phosphate. Front. Plant Sci..

[48] Wang Q., Ni J., Shah F., Liu W., Wang D., Yao Y., Hu H., Huang S., Hou J., Fu S. (2019). Overexpression of the stress-inducible *SsMAX2* promotes drought and salt resistance via the regulation of redox homeostasis in *Arabidopsis*. Int. J. Mol. Sci..

[49] Li D.D., Lin R., Li X., Zheng Y.P. (2022). Functional analysis of *AtJAR1* gene in salt tolerance of *Arabidopsis thaliana*. J. Zhejiang A&F Univ..

[50] Xie Y., Tan H., Ma Z., Huang J. (2016). DELLA Proteins Promote Anthocyanin Biosynthesis via Sequestering MYBL2 and JAZ Suppressors of the MYB/bHLH/WD40 Complex in *Arabidopsis thaliana*. Mol. Plant.

[51] Cahoon E.B., Marillia E.F., Stecca K.L., Hall S.E., Taylor D.C., Kinney A.J. (2000). Production of fatty acid components of meadowfoam oil in somatic soybean embryos. Plant Physiol..

[52] Constantine N.G., Ries S.K. (1977). Superoxide dismutases: I. Occurrence in higher plants. Plant Physiol..

[53] He X., Gao S. (2008). Changes of Antioxidant enzyme and phenylalanine ammonia-lyase activities during Chimonanthus praecox seed maturation. Z. Naturforschung C J. Biosci..

[54] Landi M. (2017). Commentary to: “Improving the thiobarbituric acid-reactive-substances assay for estimating lipid peroxidation in plant tissues containing anthocyanin and other interfering compounds” by Hodges et al., *Planta* **1999**, *207*, 604–611. Planta.

[55] Bose J., Xie Y., Shen W., Shabala S. (2013). Haem oxygenase modifies salinity tolerance in Arabidopsis by controlling K^+^ retention via regulation of the plasma membrane H^+^-ATPase and by altering SOS1 transcript levels in roots. J. Exp Bot..

